# Dorsal penile nerve block for robot-assisted radical prostatectomy catheter related pain: a randomized, double-blind, placebo-controlled trial

**DOI:** 10.1186/2193-1801-3-181

**Published:** 2014-04-07

**Authors:** Aaron C Weinberg, Solomon L Woldu, Ari Bergman, Arindam RoyChoudhury, Trushar Patel, William Berg, Christel Wambi, Ketan K Badani

**Affiliations:** Department of Urology, Columbia University Medical Center, New York, NY 10032 USA; Department of Biostatistics, Columbia University Medical Center, New York, NY 10032 USA

**Keywords:** Postoperative pain, Prostate neoplasm, Analgesia, Prostatectomy, Urinary catheterization

## Abstract

**Purpose:**

Following Robotic-Assisted Radical Prostatectomy (RARP) patients routinely have penile pain and urethral discomfort secondary to an indwelling urethral catheter. Our objective was to assess the effect of dorsal penile nerve block with bupivacaine on urethral catheter-related pain after RARP.

**Methods:**

From 2012–2013, 140 patients with organ-confined prostate cancer were enrolled in an IRB approved double-blinded, randomized control trial comparing a dorsal penile nerve block of bupivacaine versus placebo after RARP performed by a single-surgeon. Patients were asked to complete questionnaires using the Wong-Bakers FACES Pain Rating scale while hospitalized and for 9 days post-operatively, until the catheter was removed. The primary end-points were: catheter-related discomfort, abdominal (incisional) pain, and bladder spasm-related discomfort. Secondary end-points included narcotic and other analgesic usage.

**Results:**

120 patients were randomized to placebo vs. bupivacaine dorsal penile nerve bock. The two arms (n = 56 bupivacaine and n = 60 placebo) did not differ in preoperative, perioperative, or pathological results. There was no difference in narcotic utilization between the two cohorts. Abdominal pain was slightly lower in the bupivacaine arm at 6 hours compared to the placebo arm, but there was no difference in abdominal pain at other time points, and there were no differences in reported catheter-related discomfort or bladder spasm-associated discomfort at any of the measured time points.

**Conclusions:**

The data does not support the routine use of a dorsal penile nerve block with bupivacaine following RARP.

## Introduction

Over the past decade, robot-assisted radical prostatectomy (RARP) has surpassed open radical prostatectomy as the most common surgical treatment for prostate cancer. Greater than 75% of radical prostatectomies in the United States and Europe are now performed with the da Vinci Surgical System (Intuitive Surgical, Sunnyvale, CA) (Mottrie and Ficarra 2010). The rapid adoption of RARP has resulted, in part, due to a shallower learning curve as well clinical benefits including decreased blood loss, transfusion requirement, duration of hospital stay, and perioperative complication rates, without sacrificing oncologic outcomes (Ficarra et al. 2009; Barocas et al. 2010). RARP is also associated with decreased postoperative pain at the incision sites (Ficarra et al. 2009). Patients require postoperative bladder drainage, most commonly via urethral catheterization, which is associated with significant patient-reported physical limitations and discomfort (Lepor et al. 2001).

Urinary catheter-related discomfort has been described as a burning sensation spreading from the suprapubic area to the penis with an urge to void thought to be due to friction between the catheter and the urothelium. This friction causes involuntary bladder contractions through muscarinic receptors; the first line treatment is anticholinergic medications similar to the treatment for overactive bladder. However, these treatments are not without side effects, most notably dry mouth, blurred vision, and facial flushing (Kaufman et al. 2007). Targeting this discomfort early is essential, because these effects often first occur in the recovery unit, and are very concerning for patients and their families as it often exacerbates the incisional pain from surgery. Intravenous or oral narcotic medication have been shown to be effective in the prevention and treatment catheter-related discomfort, but these agents cause sedation after the operation, delayed bowel function, and may prolong hospitalization (American Society of Anesthesiologists Task Force on Acute Pain Management 2012).

Patients are routinely managed with a variety of pain medications including opiates, non-steroidal anti-inflammatory drugs, and acetaminophen while in the hospital. Upon discharge, many patients are continued on a similar cocktail of orally available medications while convalescing at home. Parenteral ketorolac has been investigated by many groups internationally and it is debated on its efficacy in preventing or decreasing catheter discomfort (Agarwal et al. 2006a). At our intuition, ketorolac is given at the discretion of the anesthesiologist, and is primarily used to minimize the amount of narcotic medication administered postoperatively. Currently there is no specific countermeasure for catheter-related discomfort. In the event of significant catheter-related bother, patients are simply reassured.

Bupivacaine is a Food and Drug Administration (FDA)-approved local anesthetic of the amide group used for peripheral nerve blocks for many urologic and general surgical procedures (Babst and Gilling 1978; Leone et al. 2008; Gauntlett 2003). The dorsal penile nerve block has been extensively studied in the setting of neonatal circumcision (Bacon 1977), and has been shown to be safe and effective (Brady-Fryer et al. 2004). There have been no studies in the literature evaluating the ability of a dorsal penile nerve block to alleviate catheter-related discomfort. Furthermore, it is not known whether diminution of catheter-related discomfort can affect the overall perception of postoperative pain following radical prostatectomy. Anecdotal evidence using a bupivacaine dorsal penile nerve block following endoscopic treatment of urethral stricture disease, with subsequent long-term urethral catheter drainage, has had beneficial results. We investigate the use of bupivacaine dorsal penile nerve block to control post-operative pain following RARP to add further techniques to the pain management armamentarium.

## Materials and methods

After obtaining the approval of the Columbia University Medical Center Institutional Review Board, we conducted a prospective, randomized, double-blinded, placebo-controlled study comparing bupivacaine (Marcaine, 5 mg/mL, Hospira Inc., Lake Forest, IL) dorsal penile nerve block versus normal saline injection at the conclusion of RARP for localized prostate cancer.

Between January 2012 and May 2013, 140 patients were initially enrolled in this study. We excluded those with chronic pain conditions, patients currently on narcotics, patients with history of indwelling-catheterization, urethral or prostatic surgery, patients allergic to bupivacaine or other local anesthetics, patients with bleeding disorders, abnormal genital anatomy or liver abnormalities. All standard FDA contraindications and warnings for bupivacaine were considered amongst our exclusion criteria. All patients signed informed consent before participating and were blinded to the randomization scheme.

All patients underwent standard RARP with bilateral pelvic lymph node dissection performed by a single surgeon (KKB). The camera trocar was placed just to the left of the umbilicus and was extended at the conclusion of the procedure to allow for specimen extraction. This site was then closed with interrupted figure-of-eight sutures. All skin incisions were infiltrated with 10–20 mL of 0.25% bupivacaine. After the urethral anastomosis was complete, but prior to the placement of the final 18-French catheter, the patient received a dorsal penile nerve block using bupivacaine or an injection of saline based on their randomization assignment. The study-coordinator, who was the only team member who knew the randomization results, handed the surgeon a syringe containing either 20 mL of bupivacaine 0.25% or 20 mL of saline (placebo) for the penile block. The nerve block was performed by anesthetizing the right and left dorsal penile nerves, the deepest divisions of the pudendal nerve (Gauntlett 2003). The syringe was aspirated to ensure that no vascular structure was entered. A total of 20 mL was injected subcutaneously in the sub-pubic space at the base of the penis, cephalad to the dorsal penile vessels, as well as circumferentially at the time of skin closure. A 20 mL syringe attached to a 21-gauge needle was used for all injections.

After the operation, blinded research assistants administered questionnaires related to the patient’s pain from three domains: abdominal (incisional) pain, urethral catheter-related pain, and bladder spasm-related discomfort. The questionnaires were based on the Wong-Baker FACES Pain Rating visual analog scale (VAS), which rates pain on a 0–10 scale (Price et al. 1983). The questionnaires were administered once the patients arrived in the post-operative recovery unit at 30 minutes, 90 minutes, 6 hours, 12 hours, 18 hours, and 24 hours post-operatively. All patients were discharged on the first post-operative day. After discharge, the patients were asked to fill out the same questionnaire regarding their pain parameters on a daily basis for 9 days until their follow-up appointment and catheter removal.

Data on analgesic and anticholinergic medication usage while in the hospital was collected via the aid of an electronic medication dispensation and recording system. Medications that were recorded included all opiate analgesics, non-steroidal anti-inflammatory medications (in the form of intravenous ketorolac) and anticholinergic medication (in the form of oral oxybutynin 5 mg).

Our standard post-operative pain regimen for patients following RARP is the following: acetaminophen 325 mg PO 1 tab every 4–6 hours for pain score 1–3 on a 10-point scale, acetaminophen/codeine 325 mg/30 mg PO 1 tab every 4–6 hours for pain score 4–6 on a 10-point scale, acetaminophen/codeine 325 mg/30 mg PO 2 tabs every 4- 6 hours for pain score 7–10 on a 10-point scale, and hydromorphone 1 mg IV every 3 hours for breakthrough pain. No patients are prescribed standing opiate medication. Patients are given standing post-operative ketorolac IV (15 mg or 30 mg) every 6–8 hours if they have normal renal function and no operative contraindication. For those who experienced bothersome bladder spasms, anticholinergic medications are given on a PRN basis in the form of oxybutynin 5 mg PO every 8 hours.

On discharge, patients are prescribed acetaminophen/codeine 325 mg/30 mg PO 1–2 tab every 4–6 hours on a PRN basis. Once at home, in addition to filling out pain questionnaires, they were also asked to self-report opiate usage on a daily basis. While the above analgesic medications and dosages represent our standards following RARP, the physicians taking care of the patient were at liberty to deviate from these standards if deemed necessary.

To facilitate analysis, all doses of opiates taken by the patient were converted to oral morphine sulfate equivalents doses (MED) (Svendsen et al. 2011). Using pain scores from similar studies in the urologic literature we assumed a VAS difference of 1.5 (on a scale of 1–10) between placebo and bupivacaine with a standard deviation of 1.3 (pooled standard deviation of 0.9 to 2.1) and a power of 99%, the sample size need for each group was 50. We estimated a dropout rate of 20% and we planned to enroll 60 patients. Student’s t-test was used to compare continuous variables and chi-square analysis was used to compare categorical distributions between the treatment groups. A post-hoc modified bonferroni correction was used to account for alpha inflation due to multiple primary end-points. Analysis was performed with SPSS version 21.0 (IBM, Armonk, NY) with p < 0.05 defined as significant.

## Results

**Figure 1:**
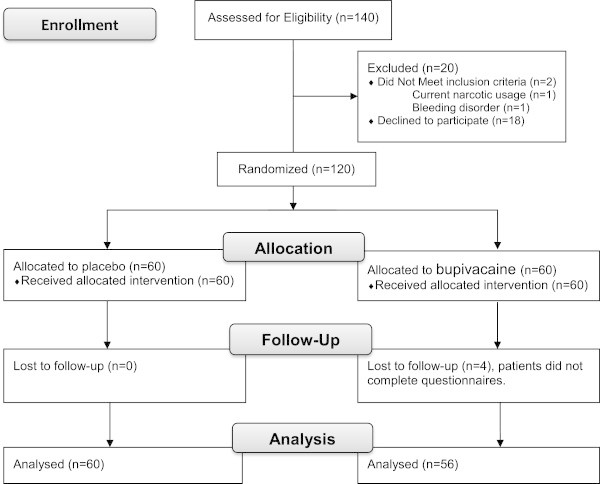
**CONSORT flow diagram.**

140 patients were initially enrolled into the study. 2 patients were excluded due to current narcotic usage (n = 1) and bleeding disorder (n = 1). 18 patients withdrew prior to randomization, leaving 120 patients who were randomized in a 1:1 fashion to either a placebo (saline) injection or bupivacaine dorsal penile nerve block. After randomization, no patients were lost to follow-up in the placebo group (n = 60) and four were lost in the bupivacaine group (n = 56), leaving 116 patients for final analysis (Figure 1). Mean patient age was 61 (range 46–75). Demographics and clinical characteristics of the cohorts were comparable; there was no difference with respect to age, body mass index (BMI), incidence of diabetes or peripheral vascular disease (PVD), preoperative PSA, stage, or pathologic Gleason score between the two arms (Table 1).

**Table 1:** **Baseline Characteristics and Intra-Operative Medication Utilization**

	Placebo penile block (n = 60)	Bupivacaine penile block (n = 56)	p-value
**Baseline clinical characteristics**			
Age (range)	60.8 (47-72)	61.2 (46-75)	0.78
BMI (range)	27.8 (18.8-40.0)	27.1 (20.1-38.5)	0.28
Diabetes (%)	4 (6.4%)	6 (10.3%)	0.74
PVD (%)	1 (1.6%)	1 (1.7%)	0.96
History of narcotics use (%)	0 (0%)	1 (3.1%)	0.48
PSA (range, ng/mL)	6.2 (0.9-24.0)	6.6 (0.9-25.7)	0.54
Operative time (mean ± SD, minutes)	186 ± 79	166 ± 80	0.18
Preoperative Gleason score			0.84
6	19 (32%)	22 (39%)	
7	34 (58%)	28 (49%)	
≥8	6 (10%)	7 (12%)	
**Intra-operative medications**			
Opiates (mean ± SD, mg MED)	94.1 ± 39.6	89.3 ± 38.2	0.51
Ketorolac usage	38%	34%	0.63

The two arms did not differ in the amount of opiate medications or ketorolac usage or dose given intraoperatively (Table 1). The mean ± SD intraoperative opiate amount given to the patients in the placebo arm was 94.1 ± 39.6 mg of MED and 89.3 ± 38.2 mg of MED in the bupivacaine arm (p = 0.51).

**Figure 2:**
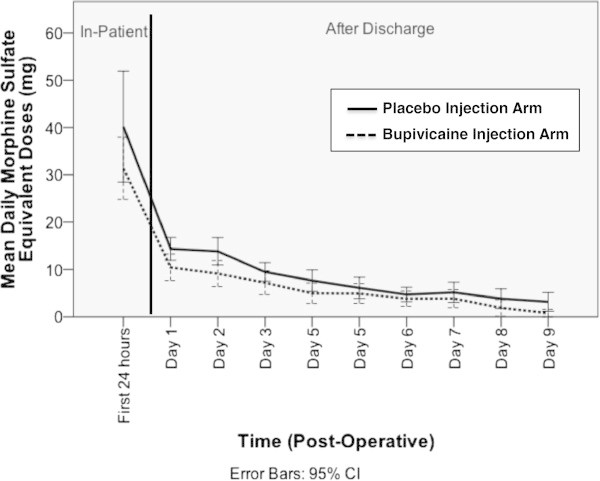
**Post-Operative Opiate Utilization Following RARP with Placebo vs. Bupivacaine Dorsal Penile Nerve Block.**

When analyzing the use of analgesic medication while in the hospital, there was no difference in the incidence of utilizing periurethral lidocaine jelly for catheter-related discomfort, anticholinergic medication (given as oxybutynin 5 mg PO) for bladder spasms, opiates usage, or ketorolac utilization or dose given. The mean ± SD inpatient opiate usage was 41.2 ± 45.5 mg for the placebo arm and 31.5 ± 24.6 mg for the bupivacaine arm (p = 0.16, Table 2). The outpatient opiate usage following discharge was calculated at the time of follow-up for catheter removal was decreased in the bupivacaine cohort (mean ± SD 68.5 ± 64.3 mg of MED) compared to the placebo cohort (47.3 ± 51.1 mg of MED), but this did not reach statistical significance with a p-value of 0.05 (Table 2, Figure 2).

**Table 2:** **Post-Operative Pain Medication Utilization**

	Placebo penile block (n = 60)	Bupivacaine penile block (n = 56)	p-value
**Inpatient medications**			
Periurethral Lidocaine Jelly Usage (%)	8%	16%	0.20
Anticholinergic, Usage (%)	28%	25%	0.73
Anticholinergic (# doses of oxybutynin 5 mg)	0.8 ± 2.1	0.5 ± 0.9	0.21
Opiates (mean ± SD, mg of MED)	41.2 ± 45.5	31.5 ± 24.6	0.16
IV Ketorolac Usage	73%	84%	0.17
IV Ketorolac (mean ± SD, mg)	49.5 ± 34.8	57.3 ± 32.5	0.62
**Post-discharge medications**			
Opiates (mean ± SD, mg of MED)	68.5 ± 64.3	47.3 ± 51.1	0.05

**Figure 3:**
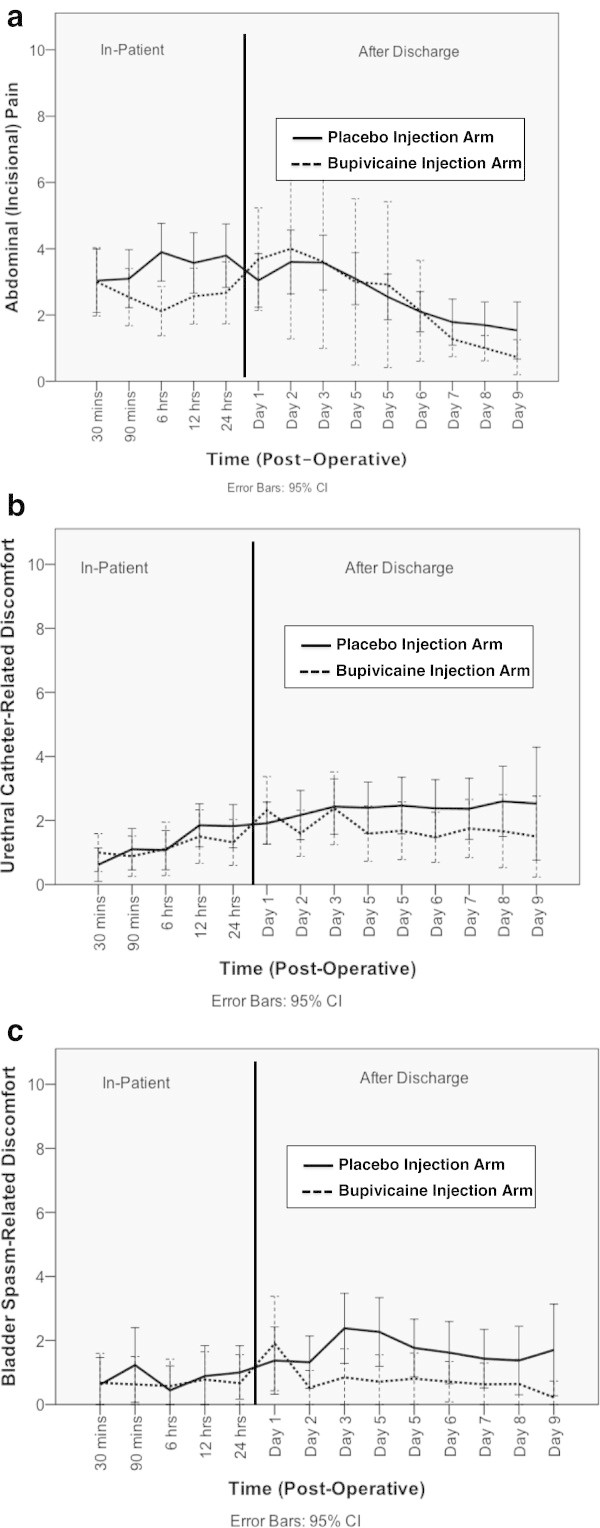
**Post-Operative Pain or Discomfort Measured by Questionnaire in Three Domains (Based on a 10-Point Wong-Baker FACES Pain Rating Scale). a**. Post-Operative Abdominal (Incisional) Pain Following RARP with Placebo vs. Bupivacaine Dorsal Penile Nerve Block. **b**. Post-Operative Urethral Catheter-Related Discomfort Following RARP with Placebo vs. Bupivacaine Dorsal Penile Nerve Block. **c**. Post-Operative Bladder Spasm-Related Discomfort Following RARP with Placebo vs. Bupivacaine Dorsal Penile Nerve Block.

When considering the subjective, patient-reported pain parameters, there was a decreased reported sensation of abdominal (incisional) pain 6 hours after the operation in the bupivacaine arm (mean ± SD score of 2.0 ± 1.9 out of 10-point scale) compared to the placebo arm (mean ± SD score of 3.7 ± 2.4) with a p = 0.004 (Figure 3a). There were no significant differences in reported abdominal pain at the other time points examined, or at any time points examined when patients were asked to report the pain or discomfort related to the urethral catheter (Figure 3b) or related to bladder spasm (Figure 3c).

No adverse events were reported by any patients receiving either saline or bupivacaine blocks, including local and allergic reactions.

## Discussion

Robot-assisted radical prostatectomy is associated with shorter duration of hospital stay and post-operative pain, but catheter-related discomfort remains a burden to patients (Ficarra et al. 2009). In designing this study, we hypothesized that the administration of bupivacaine as a regional dorsal penile nerve block would reduce post-operative pain associated with an indwelling catheter. The half-life of bupivacaine is 2.7 hours in adults, so the anesthetic effect of the penile block is in effect through the very immediate post-operative recovery period (Babst and Gilling 1978). The study was also designed to investigate whether patients that received penile nerve block would report lower pain scores during the entire duration of their recovery period, not only during the first few hours post-surgery, as pain at one site has been hypothesized to result in sensitization and lowering of pain thresholds at other sites and after the noxious stimuli has been removed (Latremoliere and Woolf 2009).

Catheter-related discomfort is a common fear discussed in pre-operative evaluation and a significant source of morbidity expressed during post-operative care following radical prostatectomy. Binhas et al. found that up to 63% of men who had an indwelling catheter post-operatively reported significant discomfort within the first hour; with men with larger catheter sizes experiencing severe discomfort two-times greater than those with smaller catheters (>18-French vs. <18-French) (Binhas et al. 2011). Additionally, Lepor at al. found that in men undergoing open radical prostatectomy greater than 50% of patients reported limitations caused by the presence of an indwelling catheter (Lepor et al. 2001).

Pain management following radical prostatectomy has largely relied on the administration of analgesic medication, particularly opiates. Several studies suggest that pain following radical prostatectomy is best controlled with ketorolac, given its efficacy for pain control, earlier return of bowel function, and association with shorter hospital stay (Kaufman et al. 2007; See et al. 1995; Kay et al. 2002). However, these studies did not distinguish between incisional and catheter-related pain. Furthermore, the effects of ketorolac on platelet function in the post-operative period have yet to be fully elucidated. In practice, ketorolac is often augmented with narcotics used to control breakthrough pain (Mazzocca et al. 2013).

The adverse effects of opioid analgesia are significant, and include nausea, pruritus, respiratory depression, and decreased intestinal motility. Decreasing post-operative opioid requirement would likely hasten the return of bowel function and shorten convalescence. Anticholinergic medications have shown to be somewhat effective at lessening post-operative opioid requirement and urinary catheter discomfort when compared to placebo (p < 0.05), but subjects still reported significant discomfort (Agarwal et al. 2006b; Tauzin-Fin et al. 2007).

Suprapubic catheters are associated with less pain and urgency than urethral catheterization following RARP (Krane et al. 2009; Orikasa et al. 2012), however many patients may object to the creation of a cystostomy for the purpose of pain control. Intravesical administration of the local anesthetic ropivicaine during RARP has been investigated, however no significant difference in reported pain scores was seen when compared to placebo (Fuller et al. 2013).

Overall, our data does not show a statistically significant difference between placebo and bupivacaine penile nerve block after RARP in the post-operative pain parameters, with the exception of the abdominal (incisional) pain at the 6 hour time-point following RARP, which was statistically significantly lower in the bupivacaine arm as compared to the placebo arm. There were also no differences in inpatient or outpatient opiate analgesic utilization between the two arms.

Despite the prospective, randomized, double-blinded, placebo-controlled study design, there are limitations to the study. It was calculated that 100 patients were required to detect a 25% decrease in reported pain scores. There may have been an overestimation of the effect of the nerve block, in which case, the study may be underpowered to detect statistically significant differences in pain scores. This theory is supported by the fact that the pain scores in both arms were fairly low. Although prospectively collected, the pain-ratings scales are still vulnerable to biases inherent in all questionnaires.

## Conclusions

Compared to placebo, the use of a bupivacaine dorsal penile nerve block does result in decreased self-reported abdominal (incisional) pain 6 hours following a RARP, however this decrease does not persist over time, nor are there significant differences in opiate utilization, urethral catheter-related discomfort, or bladder spasm-related discomfort. Overall, the study does not support the routine usage of bupivacaine dorsal penile block following RARP.

## Abbreviations

(RARP): Robot-Assisted Radical Prostatectomy
(BMI): Body Mass Index
(BCR): Biochemical Recurrence
(PSA): Prostate-Specific Antigen.
